# I-*Sce*I Endonuclease-Mediated Plant Genome Editing by Protein Transport through a Bacterial Type III Secretion System

**DOI:** 10.3390/plants9091070

**Published:** 2020-08-20

**Authors:** Yuki Yanagawa, Kasumi Takeuchi, Masaki Endo, Ayako Furutani, Hirokazu Ochiai, Seiichi Toki, Ichiro Mitsuhara

**Affiliations:** 1 Institute of Agrobiological Sciences, NARO, 1-2 Owashi, Tsukuba, Ibaraki 305-8634, Japan; yyana@affrc.go.jp (Y.Y.); kasumit@affrc.go.jp (K.T.); mendo@affrc.go.jp (M.E.); ochiaih@affrc.go.jp (H.O.); stoki@affrc.go.jp (S.T.); 2 Gene Research Center, Ibaraki University, 3-21-1 Ami, Inashiki, Ibaraki 300-0393, Japan; ayako.furutani.asano@vc.ibaraki.ac.jp

**Keywords:** bacterial type III secretion system, *Xanthomonas campestris*, I-*Sce*I, protein transportation, genome editing

## Abstract

*Xanthomonas campestris* is one of bacteria carrying a type III secretion system which transports their effector proteins into host plant cells to disturb host defense system for their infection. To establish a genome editing system without introducing any foreign gene, we attempted to introduce genome editing enzymes through the type III secretion system. In a test of protein transfer, *X. campestris* pv. *campestris* (*Xcc*) transported a considerable amount of a reporter protein sGFP-CyaA into tobacco plant cells under the control of the type III secretion system while maintaining cell viability. For proof of concept for genome editing, we used a reporter tobacco plant containing a luciferase (LUC) gene interrupted by a meganuclease I-*Sce*I recognition sequence; this plant exhibits chemiluminescence of LUC only when a frameshift mutation is introduced at the I-*Sce*I recognition site. Luciferase signal was observed in tobacco leaves infected by *Xcc* carrying an *I-SceI* gene which secretes I-*Sce*I protein through the type III system, but not leaves infected by *Xcc* carrying a vector control. Genome-edited tobacco plant could be regenerated from a piece of infected leaf piece by repeated selection of LUC positive calli. Sequence analysis revealed that the regenerated tobacco plant possessed a base deletion in the I-*Sce*I recognition sequence that activated the *LUC* gene, indicating genome editing by I-*Sce*I protein transferred through the type III secretion system of *Xcc*.

## 1. Introduction

Genome editing technology offers an attractive new approach to plant breeding. Genome editing involves making functional changes in endogenous genes by mutation and deletion with genome editing enzymes composed of protein or protein/RNA complex such as Zinc finger nucleases (ZFNs), Transcription activator-like effector nucleases (TALENs) and Clustered regularly interspaced short palindromic repeats (CRISPR)/Crispr associated protein 9 (Cas9) [1,2,3,4].

In mammalian cells, genome editing has been achieved by introducing Cas9 and guide RNA (gRNA) ribonucleoprotein complex by lipofection or electroporation [5]. In addition, microinjection was used for CRISPR/Cas9-mediated genome editing in murine zygotes [5]. Nanoparticles also worked to deliver the Cas9/gRNA complex for correcting gene mutation of genetic disease [6]. The foreign protein and RNA are degraded in the cells; therefore, genome editing is expected to accomplish functional changes without introducing any foreign gene. However, plants contain structures such as cuticular layers, wax layers, and cell walls that prevent introduction of protein and RNA directly into the cells. Thus, these methods above used in animals cannot work efficiently in intact plant cells. Due to these barriers, genome editing enzyme genes are often transformed into plant cells and removed from regenerated plants in the next generation to obtain null segregants. However, plants with long life cycles such as trees take a long time to reproduce. Moreover, crop plants cultivated by vegetative propagation such as potato and strawberry are not fixed varieties, producing a different variety to parent one in the next generation. Thus, in such kinds of plants, transformed foreign genes cannot be removed from genome-edited plants. For this reason, it has been expected to develop effective techniques to introduce genome editing enzymes as proteins and RNAs in plants.

Genome editing in tobacco BY-2 protoplasts has been achieved by using lipofection to introduce Cas9/gRNA [7]. Cell-penetrating peptides are also useful to transfer proteins into plant cells using infiltration to leaves or stem cells, although no genome-edited plant has been produced yet [8]. We previously developed a technique to introduce protein directly into plant cells by using an atmospheric-pressure plasma [9]. In this work, we suggest a new technique for protein transfer into plant cells that uses phytopathogenic bacteria. The technique was also applied to make genome-edited plants. Since bacteria can proliferate on plant tissues until suppressed by plant resistance, during proliferation, the bacteria supply genome editing enzymes to the cells of inoculated tissues while enlarging the area of infection, implying efficient genome editing. In addition, protoplast preparation is not required for bacterial infection, indicating that it is easy handling for further experiments to produce genome-edited plants.

Several pathogenic bacteria in plants and animals have delivery systems of their effector proteins into host cells named type III secretion systems to make their surroundings comfortable by controlling physiological functions of the hosts such as interfering with host immune response. If we can control the secretion system for protein carrier apparatus to the cells, it will be a useful tool for direct delivery of target proteins such as genome editing enzymes into the cells. The genus *Xanthomonas* is known as consisting of a large group of pathogenic bacteria that use the type III secretion system in plants [10,11]. Within the genus, *X. campestris* can infect a variety of host plants, implying that its secretion system could be a good candidate of protein carrier apparatus. Therefore, we tried to develop genome editing method based on protein transport through *X. campestris* pv. *campestris* (*Xcc*) into plant cells.

The purpose of this study is to develop a protein transfer method without introducing any foreign nucleic acid that can be used to produce genome-edited plants. I-*Sce*I is a homing endonuclease often used in experimental models of genome editing systems. In this work, we showed that I-*Sce*I protein was transported into tobacco leaf cells through the type III secretion system of *X. campestris* (*Xcc*) and genome-edited plants were regenerated by the *Xcc* infected leaf cells. We performed I-*Sce*I-mediated genome editing in a tobacco reporter plant in which a luciferase gene is interrupted by a I-*Sce*1 recognition site. Luciferase expression occurs only following I-*Sce*I digestion. We found that *Xcc* could transport a model protein into the cells of tobacco leaves by quantitative analysis. It was also showed that infected *Xcc* was sterilized from tobacco cells on a medium plate containing suitable antibiotics. Using this system, we successfully showed that a reporter gene in the tobacco plant was genome edited by the infection of *Xcc* carrying the *I-SceI* gene, indicating that I-*Sce*I was transported through the type III secretion system of *Xcc* into the cells of reporter tobacco leaves. This technique will be useful for plant genome editing without introducing any foreign nucleic acid, by enabling the transfer of genome editing enzymes having the abilities of both DNA binding and digestion such as TALENs.

## 2. Results

### 2.1. Protein Transport into Plant Leaf Cells by Xcc, and its Sterilization with Antibiotics

**Figure 1:**
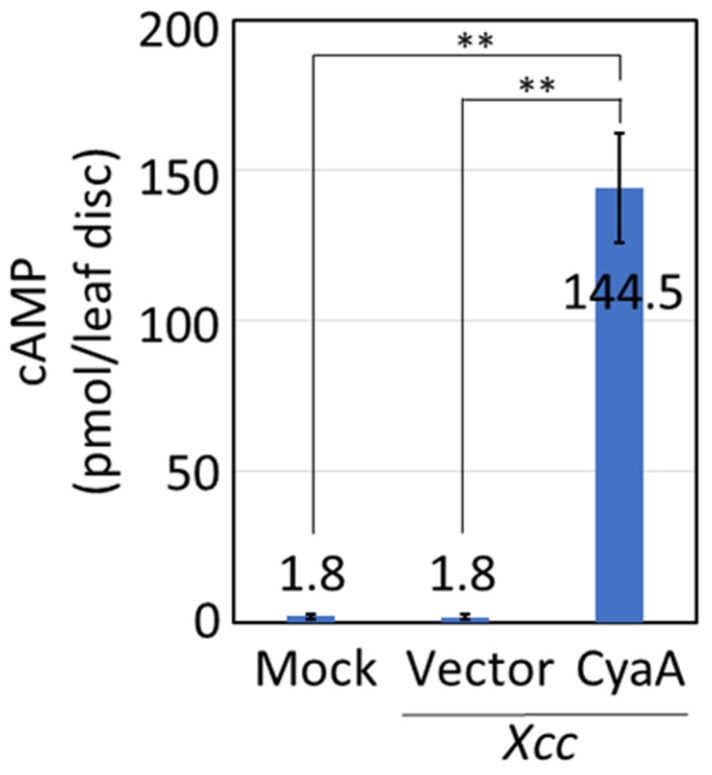
cAMP amount in tobacco leaves infected by infiltration of *Xcc.* Tobacco leaves were infected by the infiltration of *Xcc* carrying the *Xcc1_31290::CyaA* gene whose value of OD600 became 0.05. Buffer (Mock) and *Xcc* carrying an empty vector (Vector) used for *Xcc* were infiltrated as negative controls. Leaf discs were prepared after inoculation for 20 h. The amount of cAMP was measured and calculated per leaf disc. Asterisks indicate significant differences analyzed using Student’s *t*-test compared with the negative controls at *p* < 0.01 (**).

We first examined whether the type III secretion system of *Xcc* could be used to transfer proteins into plant cells. We used bacterial adenylate cyclase (CyaA) as a reporter system. CyaA, which does not exist in plants, is an enzyme to catalyze the conversion of ATP to cAMP together with endogenous calmodulin in eukaryotic cells. The amount of cAMP provides an indicator of the amount of CyaA introduced into living cells. We have previously identified 16 effector proteins in *X. oryzae* pv. *oryzae* MAFF311018 [12]. A XOO4208 ortholog named XopQ, which is one of representative effectors in *Xanthomonas*, is conserved in *Pseudomonas* and *Ralstonia*. We identified homologues of these effector proteins in the genome of *Xcc* MAFF106712 [13], and accordingly Xcc1_31290 was found to display a high level of homology (62% percent identity) to XOO4208. We decided to use a promoter and signal peptide sequence for the type III secretion system of Xcc1_31290 as an expression cassette for protein transport. Tobacco (*Nicotiana tabacum* cv. Samsun NN) leaves were infected by infiltration of *Xcc* carrying *Xcc1_31290::CyaA*, and cAMP levels were measured by an enzyme immunoassay. Tobacco leaf cells infected by *Xcc* carrying *Xcc1_31290::CyaA* contained approximately 80.3 times greater cAMP than leaf cells infected by *Xcc* carrying empty vector (Figure 1). The level of cAMP in leaf cells treated with only buffer (mock treatment) was low, similar to that in leaf cells treated with *Xcc* carrying an empty vector. These results indicate that *Xcc* transferred CyaA into the plant leaf cells through its type III secretion system.

**Figure 2:**
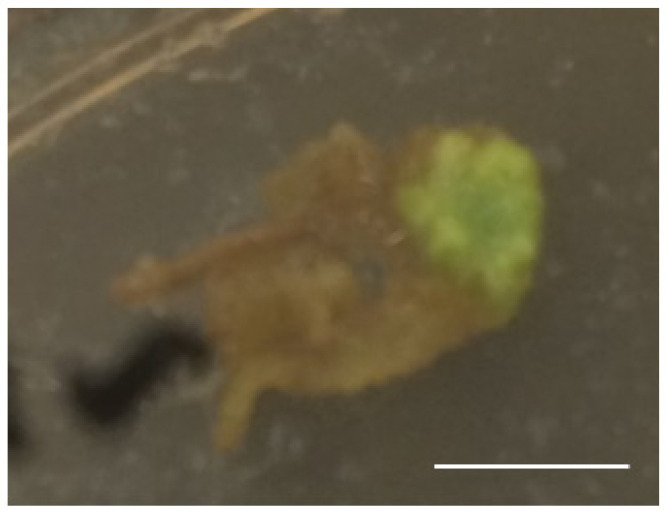
Callus from a *Xcc*-infected tobacco leaf piece. *Xcc*-infected leaf pieces were maintained on regeneration medium plates for 2 weeks. A living callus (light green) occurred from a part of a *Xcc*-infected leaf piece. Bar: 0.5 mm.

To use *Xcc* for protein transport in plant genome editing, *Xcc* must be sterilized following infection to obtain regenerated plants. To determine whether *Xcc* could be sterilized while leaving plant cells alive, *Xcc*-infected leaves were placed on regeneration medium plates containing antibiotics (200 μg/mL cefotax and 50 μg/mL rifampicin) (see Materials and Methods for detail). As shown in Figure 2, a green callus occurred from a part of the *Xcc*-infected leaves on the plate, indicating that the plant cells were alive by treatment with these antibiotics and could dedifferentiate even once *Xcc* infected them.

### 2.2. Genome Editing through I-SceI Protein Transport by Xcc into Plant Leaf Cells

**Figure 3:**
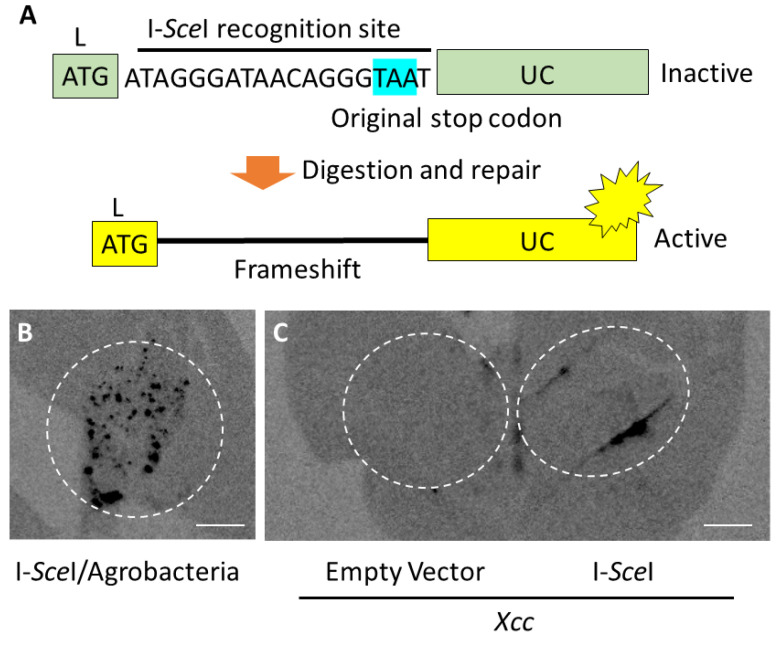
Genome editing through I-*Sce*I protein transport by *Xcc* in tabacco leaf cells. (**A**) Schematic of luciferase (LUC) activation in the leaves of an L-(I-SceI)-UC reporter tobacco plant. I-*Sce*I recognition site was inserted to divide the *LUC* gene with original stop codon. (**B**) Genome editing by infiltration of Agrobacterium carrying an *I-SceI* gene. The reporter plant with agroinfiltrated leaves was maintained for 3 days after agroinfiltration before chemiluminescence imaging. (**C**) Genome editing by infiltration of *Xcc* carrying an *I-SceI* gene or an empty vector as a negative control. The reporter plant with *Xcc* infiltrated leaves was maintained for 3 days after its infiltration before chemiluminescence imaging. Genome-edited areas are shown by dotted circles. Bars: 1 cm.

To evaluate I-*Sce*I-mediated genome editing, a transgenic tobacco plant carrying a *LUC* gene interrupted by an I-*Sce*I recognition site just downstream of the 1st ATG was produced as a reporter plant (Figure 3A). Due to this insertion, translation from the ATG codon terminates at a stop codon inside of the I-*Sce*I recognition site. If I-*Sce*I digests the DNA at the recognition site and a frameshift mutation is introduced by missrepair, the reporter gene can be repaired, leading to LUC expression.

We first examined whether this reporter system works to evaluate I-*Sce*I-mediated genome editing by *Agrobacterium*-mediated gene transfer of the *I-SceI* gene. Luciferase chemiluminescence was observed in the agroinfiltrated region (Figure 3B), indicating that the reporter system could be used to evaluate I-*Sce*I-mediated genome editing. Next, we infected reporter tobacco leaves by *Xcc* carrying the type III secretable *I-SceI* gene or an empty vector. Luciferase chemiluminescence was observed in the area infiltrated by Xcc carrying the *I-SceI* gene, but not in the area infected by Xcc carrying the empty vector (Figure 3C). This result indicates that I-*Sce*I protein produced in *Xcc* was transported into the reporter plant leaf cells, and then in-frame shift occurred in the I-*Sce*I recognition site after digestion of the target sequence by I-*Sce*I.

### 2.3. Production of Genome-Edited Tobacco Plants Through I-SceI Protein Transport by Xcc

**Figure 4:**
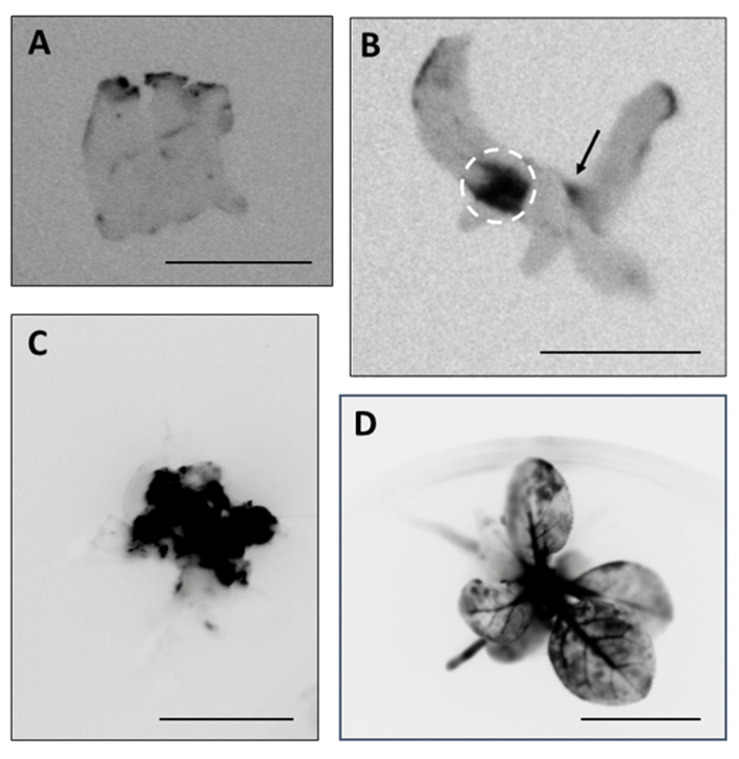
Production of genome-edited tobacco plants through I-*Sce*I protein transport by *Xcc.* (**A**) Luciferase chemiluminescence was observed in leaf pieces of an L-(I-SceI)-UC reporter tobacco plant inoculated with *Xcc* carrying the *I-SceI* gene after cultivation for 3 days on regeneration medium. (**B**) A regenerated plant was grown by selectively culturing regions that showed strong chemiluminescence. Genome-edited areas (indicated by a circle and an arrow) in B were cut out and further cultured to dedifferentiate (**C**) and differentiate (**D**). Bars: 1 cm.

To produce genome-edited plants, leaf pieces inoculated with *Xcc* containing the *I-Sce1* gene were cultivated in vitro for regeneration. *Xcc* infiltrated leaves that showed strong LUC chemiluminescence was excited and cultured on callus induction medium plates containing antibiotics cefotax and rifampicin in addition to suitable plant hormones for 1 month as described in Materials and Methods. During the dedifferentiation and differentiation from the leaf piece, areas with strong chemiluminescence were selected manually to concentrate the genome-edited cells (Figure 4A). After repeated dedifferentiation and differentiation, while concentrating area with strong chemiluminescence, on the regeneration medium plates containing cefotax, regenerated plants with genome-edited cells were produced on medium plates containing cefotax and suitable plant hormones (Figure 4B,C). By image analysis, the LUC chemiluminescence appeared patchy in the regenerated plants, indicating that they possessed genome-edited cells as mericlinal chimeras (Figure 4D).

**Figure 5:**
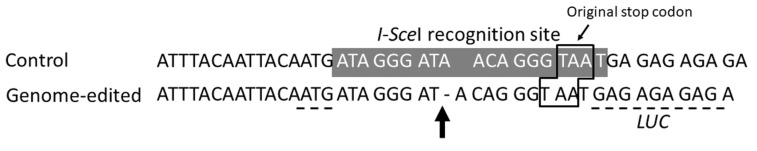
Genome sequences containing the I-*Sce*I recognition site in genome-edited and untreated control plants. Genome DNAs were prepared from L-(I-SceI)-UC leaves of plants genome-edited by *Xcc* containing the I-*Sce*I gene or untreated plants (Control). The arrow indicates a single base deletion in the I-*Sce*I recognition site.

Next, we examined the sequence in the I-*Sce*I recognition site in the reporter gene of the regenerated plant shown in Figure 4D. A fragment of the reporter gene containing the I-*Sce*I recognition site was amplified by PCR and cloned (see Materials and Methods). Ten colonies were selected, and their inserted genes were sequenced. Three out of the 10 colonies possessed inserted genes with a single base deletion in the I-*Sce*I recognition site, causing an in-frame shift of the *LUC* gene (the “Genome-edited” sequence in Figure 5); the remaining seven colonies carried inserted genes with the same sequence found in untreated control plants (“Control” sequence in Figure 5).

## 3. Discussion

In this work we demonstrated the feasibility of plant genome editing without introduction of any foreign DNA or RNA by transferring genome editing enzymes into living cells in tobacco leaves through the type III secretion system of the bacterium *X. campestris*. We showed that *Xcc* was capable of transferring a considerable amount of proteins into tobacco plant leaf cells through its type III secretion system. In addition to protein transfer, we showed that *Xcc* could be sterilized by treating *Xcc*-inoculated leaves with appropriate antibiotics while maintaining the viability of the inoculated plant cells. Finally, we demonstrated a successful application of this *Xcc* system to transferring I-*Sce*I protein into the living cells of tobacco plant leaves to produce genome-edited plants.

To evaluate protein transfer through the *Xcc* type III secretion system, we used CyaA as a reporter protein as we did previously to analyze protein introduction by plasma treatment [9]. In this previous study, the amount of cAMP produced by CyaA introduced by plasma treatment was 1.3–4.0 times greater than the amount of cAMP in a negative control; in the current study as shown in Figure 1, the amount of cAMP produced by CyaA introduced by inoculation with *Xcc* carrying *Xcc1_31290::CyaA* was 80.3 times greater than in a negative control (*Xcc* carrying an empty vector). Together, these results suggest that the *Xcc* type III secretion system achieves a higher efficiency of protein transfer than the plasma method. This higher efficiency may be due to the continuous protein transfer that occurs during *Xcc* growth in infected plant tissues, which contrasts with the one-time protein introduction by the plasma treatment. So far, there has been no report of genome editing by protein introduction into plant cells using cell-penetrating peptides, which is also one-time protein introduction [8]. The *Xcc* type III secretion system should have the advantage that it can supply protein continuously until *Xcc* sterilization, enhancing the transfer of proteins. For efficient genome editing using the *Xcc* type III secretion system, increasing efficiency of protein transport may be expected. *Xcc* probably continues to transport protein into plant cells while inoculated. Low virulent *Xcc* could be used for protein transport to increase the amount of protein transferred into plant cells by extending the period before sterilization. Promoters from other effectors in *Xcc* that may produce greater amounts of protein to increase protein transport into plant cells will be characterized in our future research.

In this work, we used tobacco plants, which belong to the *Solanaceae* family, as a model. Fortunately, *Xcc* can also infect other types of host plant species including *Brassicaceae* and *Cucurbitaceae*, making the type III secretion system of *Xcc* widely applicable to editing genomes of plants of agronomic importance. There are a lot of pathovars of *X. campestris* such as *X. campestris* pv. *carotae* and *X. campestris* pv. *juglandis* that can infect carrot and walnut, respectively. Thus, pathovars of *X. campestris* and the promoters of their effectors should be examined for efficient protein transport into the cells of various host plant species for genome editing. Other bacteria that possess the type III secretion system such as *Rhizobium* and *Pseudomonas* could also be used instead of *Xcc*.

This work suggests that the bacterial type III secretion system will be an important technique for genome editing in plants by direct introduction of genome editing enzymes into plant cells. Using this system, the type III secretion system of *Xcc* worked for I-*Sce*I mediated-genome editing by transferring I-*Sce*I protein into plant cells without introducing any foreign nucleic acid. Our model system of genome editing that used I-*Sce*I can be easily adapted to deliver artificial nucleases such as TALENs. We will try to produce genome-edited plants using TALENs by our *Xcc* system in the near future. The reporter system used in this study is useful to concentrate genome-edited cells. However, the endogenous genome does not have any selection marker, implying that increasing efficiency of genome editing is needed to produce regenerated plants carrying mutations in endogenous genome. We plan to improve our *Xcc* system to make it an effective method of plant genome editing.

## 4. Materials and Methods

### 4.1. Plant and Bacterial Materials

The *L-*(*I-SceI*)*-UC* reporter gene [14] was introduced into a tobacco plant (*Nicotiana tabacum* cv. Samsun NN). The plant was grown at 25 °C with cycles of 16 h light/8 h dark.

*Xanthomonas campestris* pv. *campestris* (*Xcc*) or *Agrobacterium tumefaciens* strain C58C1 was grown in LB medium with or without suitable antibodies with shaking at 28 °C.

### 4.2. Construction of Plasmids and Transformation into Bacteria

A plasmid expressing a fusion of the *Xcc* effector candidate and CyaA was generated as follows. We designed a pair of primers “Xcc1_31290 cya S” (5′-aagaattcgccacagaagtcactgggaagg-3′) and “Xcc1_31290 cya AS” (5′-ttggtaccctttgcgagccgggcgcgctcc-3′) including restriction enzyme sites to amplify a promoter region of *Xcc1_31290*. The PCR products were digested with *Eco*RI and *Kpn*I and were subcloned into pBluescript II KS+, and the sequence was confirmed. The fusion fragment was then cloned into pHMCyA [12] to obtain a plasmid containing the *Xcc1_31290*::*CyaA* fusion, and the plasmid was introduced into Xcc MAFF106712 by electroporation.

To construct a plasmid expressing the Xcc1_31290::I-*Sce*I fusion protein, the *Xcc1_31290* gene including the promoter region was amplified by PCR using the primers “Xcc31290FowBamI” (5′-acgtggatccacagaagtcactgggaagg-3′) and “Xcc31290Rev” (5′-gttgaacacgccctttgcga-3′). *I-SceIAtNt* was amplified by PCR using the primers “I-SceIAtNtFow” (5′-tcgcaaagggcgtgttcaacatgaagaagaagagaaagg-3′) and “I-SceIAtNtRevKpnI” (5′-acgtggtacctcatttcaaaaaggtctcac-3′). Both amplified fragments were mixed as templates for PCR using the primers “Xcc31290FowBamI” and “I-SceIAtNtRevKpnI”. The resulting Xcc1_31290::I-*Sce*IAtNt fragment was inserted into Zero Blunt^TM^ TOPO^TM^ PCR Cloning vector (Thermo Fisher Scientific, Waltham, MA, USA). The Xcc1_31290::I-*Sce*IAtNt fragment was digested with *Bam*HI and *Kpn*I and inserted into pME6031. The resulting plasmid was introduced into *Xcc* as described above.

*pCAMBIA1390-sGFP* [15] was digested with *Sal* I and *Bsr*G I to remove an open reading frame of the *sGFP* gene, and a synthesized I-*Sce* I codon-optimized for Arabidopsis and tobacco was inserted between the *Sal* I and *Bsr* G1 sites to produce pCa-HPT-I-SceIAtNt plasmid. This plasmid was introduced into *A. tumefaciens* by electroporation.

### 4.3. Infection of Bacteria and cAMP Enzyme Immunoassay

Infection of *Agrobacterium* was performed as described previously [16]. *Xcc* containing pHMCyaA or a control vector was cultivated overnight, and then harvested and suspended in A buffer (10 mM MES, 10 mM MgCl_2_, pH 5.6) at OD_600_ = 0.05 (measured using SmartSpec™ spectrophotometer, Bio-Rad, CA, USA). The suspension was infiltrated into tobacco leaves by using a needleless syringe.

To evaluate protein transport into plant cells, the amount of cAMP was measured with an Amersham cAMP Biotrak Enzymeimmunoassay System according to the manufacturer’s instruction (GE Healthcare, Tokyo, Japan). Preparation of assay sample and assay method were performed as described previously [9]. Briefly, two leaf discs of 13-mm diameter from each infected leaf were prepared with a cork borer. After the discs were ground into a powder with a mortar and a pestle with liquid nitrogen, the leaf powder was dissolved in 340 μL of 6% (*w*/*v*) trichloroacetic acid (TCA). Two-hundred microliters of assay buffer supplied by the kit were added to the dried extracts dissolved in 200 μL of the TCA homogenate. Ten microliters of each dissolved extract were used for the cAMP enzyme immunoassay. Three independent samples were used for a statistical test.

### 4.4. Genome Editing by I-SceI, Detection of LUC Chemiluminescence, and Sequencing of I-SceI Recognition Site

*Xcc* carrying an *I-SceI* gene was cultured overnight as described above. The bacteria were washed with A buffer (10 mM MES, 10 mM MgCl_2_, pH 5.6) once and diluted to OD_600_ = 0.5 with A buffer. Agrobacterium carrying an *I-SceI* gene was washed with A buffer once and diluted to OD_600_ = 0.05 with A buffer containing 150 μM 3,5′-dimetyl-4′hydroxy-acetophenone. They were infiltrated into the leaves of L-(I-SceI)-UC tobacco plants by using a needleless syringe. One millimolar of luciferin in 10 mM NaPO_4_, pH 7.0 was applied to the leaves to detect LUC chemiluminescence with a Fujifilm LAS-3000 imager (Fujifilm, Tokyo, Japan).

LUC signal was used to select genome-edited leaf regions and produce genome-edited plants. After infiltration of Xcc carrying the *I-SceI* gene, infected L-(I-SceI)-UC leaves were sterilized by soaking in 1% sodium hypochlorite for 1 min, and then washed twice with sterilized water. The leaves were cut into 0.5–1.0 cm pieces and placed on regeneration plates containing 1 × Murashige and Skoog (MS), 1 × MS vitamin (0.1 μg/mL thiamine hydrochloride, 0.5 μg/mL pyridoxine hydrochloride, 0.5 μg/mL nicotinamide, 2 μg/mL glycine, 100 μg/mL myo-inositol), 0.1 μg/mL α-Naphthaleneacetic acid, 1 μg/mL 6-Benzylaminopurine, and 200 μg/mL cefotax, and 8.5 g/L agar, pH 8.5, and were maintained at 28 °C in cycles of 16 h light/8 h dark for 3 days. The leaves were transferred onto the regeneration plates supplemented with 100 μg/mL kanamycin, 50 μg/mL rifampicin, and 30% sucrose, and leaf pieces and/or callus exhibiting strong LUC signals were transferred onto the new plates every 1 week. After 1 month, callus with strong LUC signals were transferred onto the regeneration plates with 100 μg/mL kanamycin and 30% sucrose to form shoots. The regenerated shoots were transferred onto root formation plates containing 1 × MS, 1 × MS vitamin, 30% sucrose, 200 μg/mL cefotax, and 8.5 g/L agar, pH 8.5.

To examine the sequence of the I-*Sce*I recognition site in the reporter gene of regenerated plants, a fragment of the reporter gene containing the I-*Sce*I recognition site was amplified by PCR and cloned using the primers “P35S-90” (5′-atctccactgacgtaagggatgacg-3′) and “eLUC241R” (5′-ctgcacagatcgacacgac-3′) and cloned with a Zero Blunt TOPO PCR Cloning Kit (Thermo Fisher Scientific).

## 5. Patents

A patent titled “Method for producing genome-edited plant, WO2018123938” partially resulted from the work reported in this manuscript.
